# P02 Haemophagocytic lymphohistiocytosis in mixed connective tissue disease and infection

**DOI:** 10.1093/rap/rkae117.033

**Published:** 2024-11-05

**Authors:** Stephanie Gall, Rosalind Benson

**Affiliations:** Aintree University Hospital, Liverpool, United Kingdom; Aintree University Hospital, Liverpool, United Kingdom

## Abstract

**Introduction:**

Haemophagocytic lymphohistiocytosis (HLH) is a rare, severe multisystem inflammatory condition which is associated with connective tissue disease, specifically SLE. It can be triggered by a flare in the underlying disease or infection. With a high mortality rate, early recognition and treatment is necessitated. There are few reported cases in mixed connective tissue disease (MCTD).

We present a case of HLH in a 37-year-old female with MCTD, triggered by a SLE-like flare and subsequent pneumococcal bacteraemia. The case highlights the diagnostic challenges posed by HLH in the context of concurrent infection and MCTD, and discusses the management strategies employed.

**Case description:**

A 37-year-old patient with MCTD (ANA, anti-RNP) presented to clinic with a lupus-like flare. Features included: fatigue; myalgia; aphthous ulceration; pleurisy; a low C3 and C4; raised ALT and CK. Leucopenia, neutropenia and normal renal function were noted. She had been previously stable on hydroxychloroquine and azathioprine for two years following a planned pregnancy. Longstanding disease features include telangiectasia, sclerodactyly, Raynaud’s and digital pitting. She was started on a short course of oral prednisolone with close follow up.

Two weeks later she presented with a one-day history of fever, dry cough and coryzal symptoms. CRP was acutely raised (240 mg/L), and her neutrophils and white cell count were raised. Chest X-ray showed consolidation in left upper zone and blood cultures grew streptococcus pneumoniae. Due to a penicillin allergy, she received intravenous (IV) teicoplanin and clarithromycin. Despite initial resolution of fever with clinical and biochemical improvement, at day five she developed high grade pyrexias and a new neutropenia. She was switched to meropenem, and a repeat infection screen was undertaken. She developed a classical salmon coloured, maculo-papular rash to her back and developed pancytopenia with a haemoglobin of 78 g/L, platelets of 49x10^9L and a neutrophil count of 0.5x10^9/L. Ferritin was 9037 ug/L (peak 12730). It was also noted that her AST was raised at 156, fibrinogen was low (1.32 g/L) and triglycerides were raised (5.9 mmol/L). A subsequent HLH screen revealed an H Score of 213 with an HLH probability of 93-96%.

HLH was suspected. Pulsed IV methylprednisolone was started but in view of a worsening pancytopenia and ongoing pyrexia, anakinra was added. There was rapid improvement and she was subsequently discharged. Anakinra was discontinued after 14 days, alongside reducing oral prednisolone with the planned addition of tacrolimus to her immunosuppression. The patient had expressed an ongoing desire for future pregnancies.

**Discussion:**

HLH is a rare but severe hyperinflammatory syndrome characterised by excessive activation of the immune system. It can occur in the context of various conditions, including infections, malignancies, and autoimmune diseases. HLH is well recognised in SLE; however, there are few reports of this in MCTD. HLH in MCTD has been reported when a patient has SLE-like clinical features.

In both HLH and flares of MCTD with SLE features, you may find cytopenia, fever, hepatosplenomegaly, lymphadenopathy, and signs of multi-organ involvement. Consequently, a high index of suspicion and careful assessment is required to elucidate key features enabling prompt treatment. Furthermore, a history of infection further complicates the picture.

Key clinical features helped us elucidate a diagnosis of HLH in this case. She had a classical rash associated with fever. The profound and acute cytopenias, alongside the elevated inflammatory markers such as ferritin and triglycerides, are critical signs pointing towards HLH. Despite initially presenting with fever associated with her pneumococcal bacteraemia, she had significantly improved prior to the return of her fever alongside the additional development of a rash. A repeat septic screen including further multiple negative blood cultures, negative extensive virology, a computed tomography chest, abdomen and pelvis (CT CAP) showing no evidence of deep-seated infection and a normal lumbar puncture, resulted in our conclusion that infection was unlikely to be the cause of her fever.

Typically, it would be expected that cytopenias related to a lupus flare would show some improvement with oral and then subsequent IV methylprednisolone. The lack of response highlighted the overwhelming inflammatory picture driven by HLH which required more targeted treatment with anakinra.

**Key learning points:**

HLH in the context of MCTD with lupus-like features is a complex and potentially life-threatening condition. Clinicians must be vigilant for signs of HLH in patients with autoimmune diseases, as early diagnosis and prompt treatment are essential for improving prognosis. Infection can be a trigger in these conditions. The management strategy involves addressing both the acute hyperinflammatory state and the underlying autoimmune disorder to prevent recurrence and ensure long-term disease control.

**Learning objectives:**

• Understanding which features are consistent with HLH and how to recognise this condition early.

• Understanding the management of HLH.

• Understanding why HLH is more common in those with autoimmune conditions, especially mixed connective tissue disease.

**Key learning points:**

• Complexity of diagnosing HLH in an autoimmune context and utilising the HLH 2004 diagnostic criteria.

HLH can present with symptoms overlapping those of autoimmune diseases, making diagnosis challenging. Recognising the distinctive signs of HLH by utilising the 2004 H score and diagnostic criteria can help with the diagnosis.

• Ineffectiveness of high-dose steroids alone and the role of immunomodulatory therapy.

The patient’s lack of response to high-dose steroids highlighted the need for alternative or additional therapies. Persistent cytopenias despite aggressive steroid treatment indicated that HLH was the driving pathology rather than a lupus flare. The initiation of anakinra, an IL-1 receptor antagonist, led to significant clinical improvement, including stabilisation of cell counts and resolution of fever. This underscores the importance of cytokine-targeted therapies in managing HLH.

• Infection as a trigger.

The case demonstrates how infections (in this instance, streptococcus pneumonia) can act as a trigger for HLH in patients with underlying autoimmune diseases. Managing the infection is critical, but attention must also be given to the hyperinflammatory state of HLH.

